# Effects of Water Availability on Leaf Trichome Density and Plant Growth and Development of *Shepherdia ×utahensis*

**DOI:** 10.3389/fpls.2022.855858

**Published:** 2022-05-18

**Authors:** Ji-Jhong Chen, Youping Sun, Kelly Kopp, Lorence Oki, Scott B. Jones, Lawrence Hipps

**Affiliations:** ^1^Department of Plants, Soils, and Climate, Utah State University, Logan, UT, United States; ^2^Department of Plant Sciences, University of California, Davis, Davis, CA, United States

**Keywords:** drought tolerance, leaf reflectance, pubescence, xeric plant, water potential

## Abstract

Many arid lands across the globe are experiencing more frequent and extreme droughts due to warmer temperatures resulting from climate change, less predictable precipitation patterns, and decreased soil moisture. Approximately 60–90% of household water is used for urban landscape irrigation in the western United States, necessitating the establishment of landscapes using drought-tolerant plants that conserve water. *Shepherdia ×utahensis* (hybrid buffaloberry) is a drought-tolerant plant with dense leaf trichomes (epidermal appendages) that may limit excessive water loss by transpiration. However, little is known about how *S. ×utahensis* regulates leaf heat balance when transpirational cooling is limited. The objective of this research was to investigate the effects of substrate water availability on plant growth and development and trichome density of *S. ×utahensis*. Ninety-six clonally propagated plants were grown using an automated irrigation system, and their substrate volumetric water contents were controlled at 0.05–0.40 m^3^·m^−3^ for 2 months. Results showed that water stress impaired plant growth and increased the proportion of visibly wilted leaves. *Shepherdia ×utahensis* acclimates to drought by reducing cell dehydration and canopy overheating, which may be accomplished through decreased stomatal conductance, smaller leaf development, leaf curling, increased leaf thickness, and greater root-to-shoot ratio. Leaf trichome density increased when stem water potential decreased, resulting in greater leaf reflectance of visible light. Cell and leaf expansion were restricted under water stress, and negative correlations were exhibited between epidermal cell size and trichome density. According to our results, plasticity in leaves and roots aids plants in tolerating abiotic stresses associated with drought. Acclimation of *S. ×utahensis* to water stress was associated with increased trichome density due to plasticity in cell size. Dense trichomes on leaves reflected more lights which appeared to facilitate leaf temperature regulation.

## Introduction

Hotter and drier climates globally, coupled with periodic drought, often necessitate large quantities of irrigation water to maintain visual quality, growth, and development of landscape plants (Mee et al., 2003). Approximately 60–90% of household water is used for urban landscape irrigation in the western United States (Sovocool et al., 2006; Center for Water-Efficient Landscaping, 2020; Arizona Department of Water Resources, 2022). However, due to the increasing water demand of a growing population, designing landscapes with drought-tolerant adaptive plants or plants native to arid and semiarid areas is important for long-term water conservation in the western United States. In addition, landscape plants are threatened by increasingly common droughts and heatwaves in the western United States because they are largely reliant on irrigation (Miller et al., 2020). A recent drought caused urban vegetation coverage in downtown Santa Barbara, California, to decline from 45 to 35% (Miller et al., 2020). Hence, landscape plants characterized by morphological and physiological plasticity, which can better acclimate to water and heat stresses, are desirable for future landscapes. Unfortunately, drought responses of landscape plants are seldom investigated, and drought tolerance studies have largely been conducted based on local precipitation rates, rather than well-controlled inputs (Meyer et al., 2009).

Reduction in soil water availability causes cell dehydration, resulting in leaf wilting and degrading aesthetic appearance (Zollinger et al., 2006). Cell dehydration then prevents chlorophyll production and photosynthesis, which reduces leaf greenness and plant growth (Ahluwalia et al., 2021). For instance, *Orthosiphon aristatus* (cat’s whiskers) exhibited wilted leaves and reduced leaf and root biomass when no irrigation was applied (Kjelgren et al., 2009). Water stress also inhibits leaf expansion, reducing light-capture area (Dale, 1988) and may indirectly induce heat stress in plants because of reduced transpirational cooling to counter absorbed radiation (Nobel, 2009). *Gaillardia aristata* (blanketflower) and *Penstemon barbatus* (golden-beard penstemon), for example, showed over 50% of the leaves burned when water was limited (Zollinger et al., 2006). High temperatures may disrupt plant metabolism and protein stability, leading to leaf burn and necrosis (Taiz et al., 2015).

Plant acclimation involves changes in morphology and physiology without genetic modification (Taiz et al., 2015). Under drought conditions, plants may acclimate to drought by decreasing water loss and reducing heat load and leaf temperature (Mee et al., 2003). Root growth may be promoted to increase water uptake, leading to a greater root-to-shoot ratio (Ahluwalia et al., 2021). Water loss may be minimized via stomatal closure, leaf senescence, and reduced leaf size (Zollinger et al., 2006). For instance, Stromberg (2013) found that xeric species growing in the southern United States have greater root-to-shoot ratios, but smaller leaves, than mesic species. In hot and arid environments, plants gradually reduced their stomatal conductance and transpiration along with increasing leaf temperatures and higher leaf-to-air vapor pressure deficit (VPD) to prevent excessive water loss (Roessler and Monson, 1985). Minimizing stomatal conductance when solar radiation and air temperature are greatest at midday can protect plants from xylem dysfunction and maintain water status (Zhang et al., 2013).

Plant leaf temperature may be regulated by adjusted leaf size, orientation, and trichome density (Ehleringer et al., 1976; Nilsen, 1987; Nobel, 2009). For example, small leaves are advantageous for increasing sensible heat loss. The leaves of native plants in the western United States, such as *Artemisia tridentata* (big sagebrush) and *Cercocarpus montanus* (alder leaf mountain mahogany), are less than 2.5 cm wide, helping to reduce plant heat load more efficiently (Mee et al., 2003). Leigh et al. (2017) reported that plants in hot and dry environments of Australia, such as *Banksia grandis* (bull banksia), *Grevillea agrifolia* (blue grevillea), and *Telopea speciosissima* (waratah) have leaves covered by dense trichomes and vertical leaf orientation, which reduces the interception of solar radiation.

Trichome density has been found to be affected by soil water content, air temperature, and VPD (Ehleringer, 1982; Banon et al., 2004; Bickford, 2016; Shibuya et al., 2016). For instance, the trichome density of *Lotus creticus* (cretan trefoil) increased when the amount of irrigation water decreased by 70% (Banon et al., 2004). Shibuya et al. (2009) discovered that *Cucumis sativus* (cucumber) had 255 trichomes per cm^2^ of leaf area at a vapor pressure deficit of 0.4 kPa which increased to 463 trichomes per cm^2^ at 3.8 kPa. Ehleringer (1982) observed that trichome density of *Encelia farinosa* (brittlebush) grown in California positively correlated to the mean maximum air temperature of the growing habitat. However, the effect of water stress on plant trichome development has not been widely studied. Early research suggested leaf trichome production was promoted under water deficit (Quarrie and Jones, 1977). However, this finding contradicts the fact that plant cell division is inhibited under drought stress conditions (Dale, 1988). Brodribb et al. (2013) reported that changes in cell size provided a substantial means to modify leaf function without disturbing other tissue/organ functions. Murphy et al. (2014) found that epidermal cell expansion facilitated the decrease of stomatal density under shade, where large leaves had low stomatal density. Stomata and trichomes are both epidermal appendages and their development occurs prior to cell expansion. Hence, changes in cell size may modify trichome density under water stress.

*Shepherdia ×utahensis* ‘Torrey’ (hybrid buffaloberry) is an interspecific hybrid between *Shepherdia argentea* (silver buffaloberry) and *Shepherdia roundifolia* (roundleaf buffaloberry). *Shepherdia argentea* tolerates a wide range of growing conditions from wet to dry soil (Mee et al., 2003), while *S. roundifolia* is extremely resistant to hot and arid conditions (Sriladda et al., 2016). Xeric *S. roundifolia* has denser leaf trichomes as compared to riparian *S. argentea* (Sriladda et al., 2016), which indicates trichome density of *Shepherdia* species may be influenced by water availability. *Shepherdia ×utahensis* has leaf trichomes (Sriladda et al., 2016) and grows well in a variety of substrates (Chen et al., 2021). However, the effects of soil moisture level on trichome density have rarely been investigated. The hypotheses of this research are (1) the morphology and physiology of *S. ×utahensis* change at different substrate water contents, and (2) leaf trichome density is affected by cell size under drought. To test these hypotheses, the objectives of this research were (1) to evaluate the morphological and physiological responses of *S. ×utahensis* under various substrate volumetric water contents in a greenhouse and (2) to quantify the relationship between trichome density and water deficit.

## Materials and Methods

### Plant Materials

Cuttings were collected from *S. ×utahensis* ‘Torrey’ clone plants at the Utah Agricultural Experiment Station’s (UAES) Greenville Research Farm (North Logan, UT) on 16 July 2019 and propagated using the method of Chen et al. (2020). On 1 October 2019, rooted cuttings were transplanted to cone-trainers (D40H; Stuewe and Sons, Tangent, OR) and filled with perlite (Hess Perlite, Malad City, ID). All plants were kept in a UAES’s hoop house (Logan, UT) and irrigated with tap water (electrical conductivity = 0.36 dS·m^−1^, pH = 7.73).

On 8 October 2020, 96 plants of uniform height and shoot number were transplanted to 7.6-L injection-molded polypropylene containers (No. 2B; Nursery Supplies, Orange, CA) using a soilless substrate (Metro-Mix® 820; Sun Gro Horticulture, Agawam, MA). Plants were manually irrigated to container capacity and subsequently irrigated using a capacitance-sensor-based automated system (Nemali and van Iersel, 2006; van Iersel et al., 2010; Zhen et al., 2014) in a UAES’s research greenhouse (Logan, UT). The experiment had three blocks (replicates) and eight volumetric water content treatments at 0.05, 0.10, 0.15, 0.20, 0.25, 0.30, 0.35, and 0.40 m^3^·m^−3^ with four replications in each treatment. Within each block, 32 plants were randomly assigned to the eight treatments, and a capacitance sensor (ECH_2_O 10HS; Meter Group, Pullman, WA) was vertically inserted into the substrate (15 cm deep) of one randomly selected container in each treatment to measure substrate water content. Twenty-four soil moisture sensors were connected to a multiplexer (AM 16/32B; Campbell Scientific, Logan, UT) connected to a datalogger (CR1000X; Campbell Scientific). The datalogger was programmed to measure sensor output voltage every 15 s, and the output voltage was converted to substrate volumetric water content (θ_v_) using a substrate-specific calibration equation [ $\theta_{v}=\mathrm{voltage}\times 0.0009-0.3688$ (*r*^2^ = 0.97, *p* < 0.0001)]. Two relay controllers (SDM-CD16AC; Campbell Scientific) were connected to 24 normally-closed, 24-volt-AC solenoid valves (CPF100; Rainbird, Azusa, CA) to control the irrigation of the four plants in each treatment. The datalogger was programmed to open the solenoid valves for 5 s when the substrate volumetric water content measured by the capacitance sensor fell below the setpoint. Each plant in the capacitance-sensor automated irrigation system was irrigated using a pressure-compensated drip emitter with a flow rate at 1.3 ± 0.2 (mean ± SD) ml·s^−1^. For establishment, plants in each treatment were irrigated at the threshold of 0.35 m^3^·m^−3^ for 26 days following the protocol of Cai et al. (2012). On 15 October 2020, plants were top-dressed with a controlled-release fertilizer (Osmocote Plus 15-9-12; Israel Chemicals, Tel Aviv-Yafo, Israel) at a rate of 0.02 g·cm^−2^. On 5 November 2020, plants were inoculated with 50 ml soil collected from the rhizosphere of a *S. ×utahensis* ‘Torrey’ plant (lat. 41°45′ N, long. 111°48′ W) growing at the UAES’s Greenville Research Farm. On 13 November 2020, the experiment was initiated, and each sensor was randomly assigned to one of eight irrigation setpoints ranging from 0.05 to 0.40 m^3^·m^−3^. The substrate was gradually dried down and maintained at each setpoint until the experiment was terminated on 12 January 2021.

A substrate-specific water retention curve was established using the van Genuchten model (van Genuchten, 1980) with measurements using Tempe cells (ICT international, Armidale, Australia) and a soil water potentiometer (WP4C; Meter Group) in the Utah State University (USU) Environmental Soil Physics Laboratory (Logan, UT). Our water retention curve was similar to the results of van Iersel et al. (2013), where substrate volumetric water content at 0.40, 0.35, 0.30, 0.25, 0.20, 0.15, 0.10, and 0.05 m^3^·m^−3^ was equivalent to corresponding substrate matric potentials of −0.008, −0.012, −0.019, −0.034, −0.067, −0.159, −0.540, and −4.358 MPa, respectively. On 11 January 2021, substrate volumetric water content in each container (θ_p_) was estimated using a handheld soil moisture sensor (Hydro Sense; Campbell Scientific). The sensor output was converted to θ_p_ using a substrate-specific calibration [ $\theta_{p}=0.2923\times \mathrm{output}+0.3855$ (*p* < 0.0001; *r*^2^ = 0.99)]. The θ_p_ was converted to soil matric potential using the substrate-specific water retention curves.

### Greenhouse Environment

The average air temperature within the greenhouse was 24.7 ± 0.4°C (mean ± SD) during the day and 21.5 ± 0.3°C at night. Supplemental light was provided using 1,000-W high-pressure sodium lamps (Hydrofarm, Petaluma, CA) at a light intensity of 287.1 ± 1.4 μmol·m^−2^·s^−1^ at plant canopy level. Lamps were turned on from 0600 to 2,200 HR when greenhouse light intensity fell below 500 μmol·m^−2^·s^−1^. The daily light integral and photosynthetic photon flux density at the plant canopy level was 27.2 ± 2.4 mol·m^−2^·d^−1^ and 316.2 ± 30.4 μmol·m^−2^·s^−1^, respectively, recorded using a full-spectrum quantum sensor (SQ-500-SS; Apogee Instruments Logan, UT).

### Data Collection

To compare plant responses to different substrate volumetric water contents (van Iersel et al., 2010; Zhen et al., 2014), all parameters were measured once during the end of the experiment, except the proportion of visibly wilted leaves.

### Proportion of Visibly Wilted Leaves, Leaf Greenness, and Gas Exchange Responses

The proportion of visibly wilted leaves was graded weekly based on the percentage of wilting leaves of the canopy (Zollinger et al., 2006). Plants were rated on a scale of 1–5, where 1 = over 65% of leaves wilted; 2 = 35–65% of leaves wilted; 3 = up to 35% of leaves wilted; 4 = less than 10% of leaves wilted; and 5 = plant fully turgid (Zollinger et al., 2006). A chlorophyll meter [Soil Plant Analysis Development (SPAD)-502; Minolta Camera, Osaka, Japan] was used to record relative chlorophyll content on 6 January 2021. Five mature leaves were randomly selected from each plant for measurement, and the average value was recorded.

On 6 January 2021, midday leaf-to-air VPD, stomatal conductance, transpiration rate, and net assimilation rate were recorded using a portable photosynthesis system (CIRAS-3; PP Systems, Amesbury, MA) with a PLC3 universal leaf cuvette in a sunny day from 1,000 and 1,400 HR. A fully expanded, mature leaf was randomly selected from each plant. Steady-state gas exchange rates were recorded after the leaf was enclosed in the cuvette for approximately 1 min, in which stomatal conductance did not change in response to cuvette ambient conditions (Bunce, 2016). Within the cuvette, photosynthetic photon flux density was set at 1,000 μmol·m^−2^·s^−1^ with 38% red, 37% green, and 25% blue light provided from light-emitting diodes, whereas CO_2_ level and leaf temperature were controlled at 400 μmol·mol^−1^ and 25°C, respectively.

### Plant Growth and Water Potential

On 7 January 2021, plant height was recorded from the substrate surface to the highest shoot tip. Canopy width and length were measured in perpendicular directions. Number of shoots longer than 5 cm was recorded. Plant growth index (PGI) [(height + length + width)/3] was calculated. On 11 January 2021, four mature leaves were sampled from the second to the fifth node counting downward from the tip of the main shoot to determine leaf curling index (Nilsen, 1987). Distance between leaf margins was recorded when the leaf was flattened (D_max_) and curled (D_i_), and leaf curling index was calculated using the equation: (D_max_-D_i_)/D_max_.

Stem water potential was measured at noon using a pressure chamber (PMS Instrument Company, Albany, OR) on 11 January 2021. Five plants were randomly chosen from each treatment, except for plants at substrate volumetric water content of 0.05 m^3^·m^−3^, from which only two plants were selected due to high mortality. Stems from the outer canopy were collected, wrapped with wet paper towels, stored in zip lock bags, and placed in an insulated cooler with ice. Measurements were taken immediately after the stems were collected.

On 12 January 2021, plants were destructively harvested. Leaf number and the fresh weight (FW) of leaves and stems were recorded. The total leaf area was measured using a leaf area meter (LI-3100; LI-COR Biosciences, Lincoln, NE), and the average leaf size of each plant was calculated as the ratio of total leaf area to the number of leaves. Roots were harvested and washed with deionized water. The number of nodules was recorded. Leaves, stems, and roots were dried in an oven at 80°C for 7 days, and the dry weight (DW) was recorded. Specific leaf area (SLA) was calculated as the ratio of leaf area to leaf DW, and the root-to-shoot ratio was calculated using the dry weight of roots and shoots (leaves and stems). The water content of leaves and stems was calculated using the equation: [(FW−DW)/FW × 100% (Zhou et al., 2021).

### Leaf Reflectance and Environmental Scanning Electron Microscope Imagery

On 12 January 2021, images of the upper surface of leaves of plants at substrate volumetric water content of 0.10 and 0.40 m^−3^·m^−3^ were recorded using a dissecting microscope (BX52; Olympus, Tokyo, Japan) before plants were destructively harvested. Three plants at substrate volumetric water content of 0.10, 0.20, 0.30, or 0.40 m^3^·m^−3^ were randomly chosen and three mature leaves were sampled from the third to fifth nodes counting downward from the tip of the main shoot of each plant. Leaf size was also recorded. Leaves were stored in Petri dishes containing wet germination paper. The Petri dishes were sealed using parafilm and stored in a cooler with ice. A disk from each leaf was sampled using a #12 cork borer (diameter = 3 cm) with an area of 7 cm^2^ to study the leaf reflectance using a spectroradiometer (Apogee Instruments). The mean reflectance of photosynthetically active radiation (PAR) was calculated using the wavelengths from 400 to 700 nm. The reflectance of blue, green, and red light was calculated using the wavelengths of 450, 530, and 660 nm, respectively (Kusuma et al., 2020).

Following leaf reflectance measurements, leaf disks were immediately sent to the USU Microscopy Core Facility (Logan, UT). A sample (diameter = 0.3 cm) was collected from each leaf disk using a hole punch (McGill, Marengo, IL). Nine fields of view (0.32 mm^2^) at ×300 magnification were photographed from the upper (adaxial) surface of each leaf punch using an environmental SEM (ESEM; Quanta FEG 650; FEI Company, Hillsboro, OR). Fine-scale morphological traits were determined following the method of Murphy et al. (2016). Trichome density (trichome number mm^−2^), uncovered stomata (visible stomata mm^−2^), trichome radius (μm), trichome coverage fraction [(area covered by trichomes)/(total image area)], epidermal cell size (μm^2^), and epidermal cell density (epidermal cells·mm^−2^) were quantified in each field of view using ImageJ (Schneider et al., 2012). The values of fine-scale morphological traits from the nine fields of view were averaged for each leaf, and the mean value of three leaves was recorded for each plant. The numbers of epidermal cells and trichomes per leaf were calculated using the density and leaf size, and the ratio between trichomes and epidermal cells of each leaf was determined.

### Data Analysis

The experiment was arranged in a randomized complete block design with eight treatments and three blocks. A mixed model analysis was performed to test the effects of substrate volumetric water contents on all measured parameters. Trend analyses were conducted for all data to test the nature of the relationship between plant responses and substrate volumetric water contents. Correlation analyses were performed to study the relationships between trichome density and leaf size, epidermal cell size, epidermal cell density, or light reflectance; between leaf size and epidermal cell size or epidermal cell density; and between stem water potential and epidermal cell size. All statistical analyses were performed using PROC MIXED or PROC REG procedure in SAS Studio 3.8 (SAS Institute, Cary, NC) with a significance level specified at 0.05.

## Results

### Substrate Matric Potential

**Figure 1:**
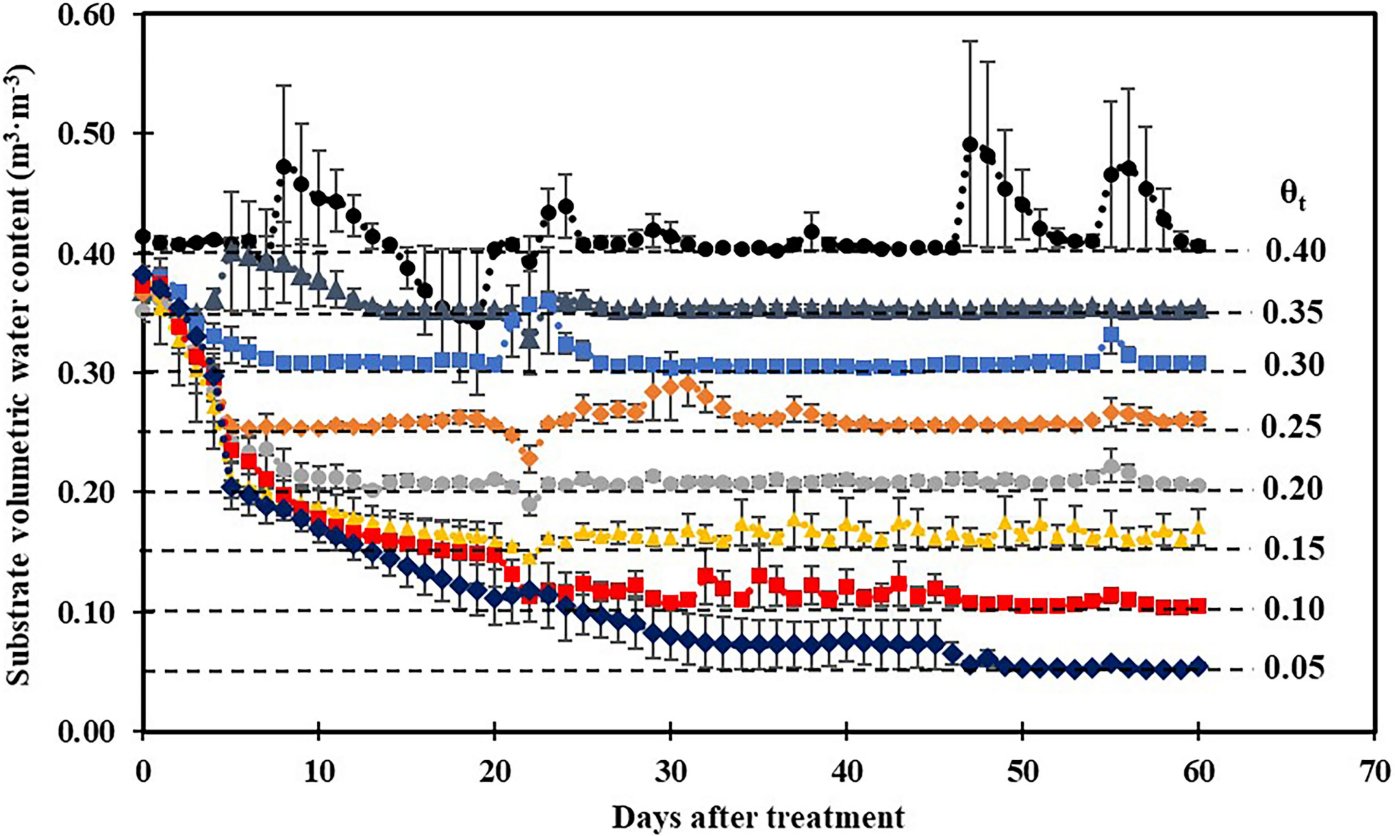
Daily average substrate volumetric water content at eight substrate volumetric water content treatments (θ_t_) recorded using calibrated soil moisture sensors (ECH_2_O 10HS; Meter Group, Pullman, WA) during the experiment. Error bars represent SEs of three sensors.

Substrate volumetric water contents were maintained well above their irrigation setpoints 30 days after treatment (Figure 1), and a cubic curvilinear relationship was observed between substrate matric potential and substrate volumetric water contents at the end of the experiment and ranged from −0.89 to −0.03 MPa when substrate volumetric water content increased from 0.05 to 0.40 m^3^·m^−3^ (Table 1).

**Table 1:** Substrate matric potential (ψ_m_) at eight substrate volumetric water content treatments (θ_t_) recorded on 11 January 2021.

θ_t_ (m^3^·m^−3^)	ψ_m_^z^ (MPa)
0.40	−0.08 a^y^
0.35	−0.03 a
0.30	−0.04 a
0.25	−0.23 ab
0.20	−0.26 ab
0.15	−0.56 bc
0.10	−0.51 b
0.05	−0.89 c
Linear	NS^x^
Quadratic	*
Cubic	****

z ψ_m_ was calculated from measurements of a handheld soil moisture sensor (Hydro Sense; Campbell Scientific) using a substrate-specific water retention curve estimated using the van Genuchten equation (van Genuchten, 1980), of which the residual water content is 0, the saturated water content is 0.74 m^3^·m^−3^, the inverse of the air entry suction is 771.43 MPa^−1^, and the dimensionless pore-size distribution is 1.33.

y Means with same lowercase letters are not significantly different among treatments by Tukey–Kramer method with a significance level specified at 0.05.

x NS, *, **** Nonsignificant, significant at *p* ≤ 0.05 or 0.0001, respectively.

### Proportion of Visibly Wilted Leaves, Mortality, and Plant Growth

**Figure 2:**
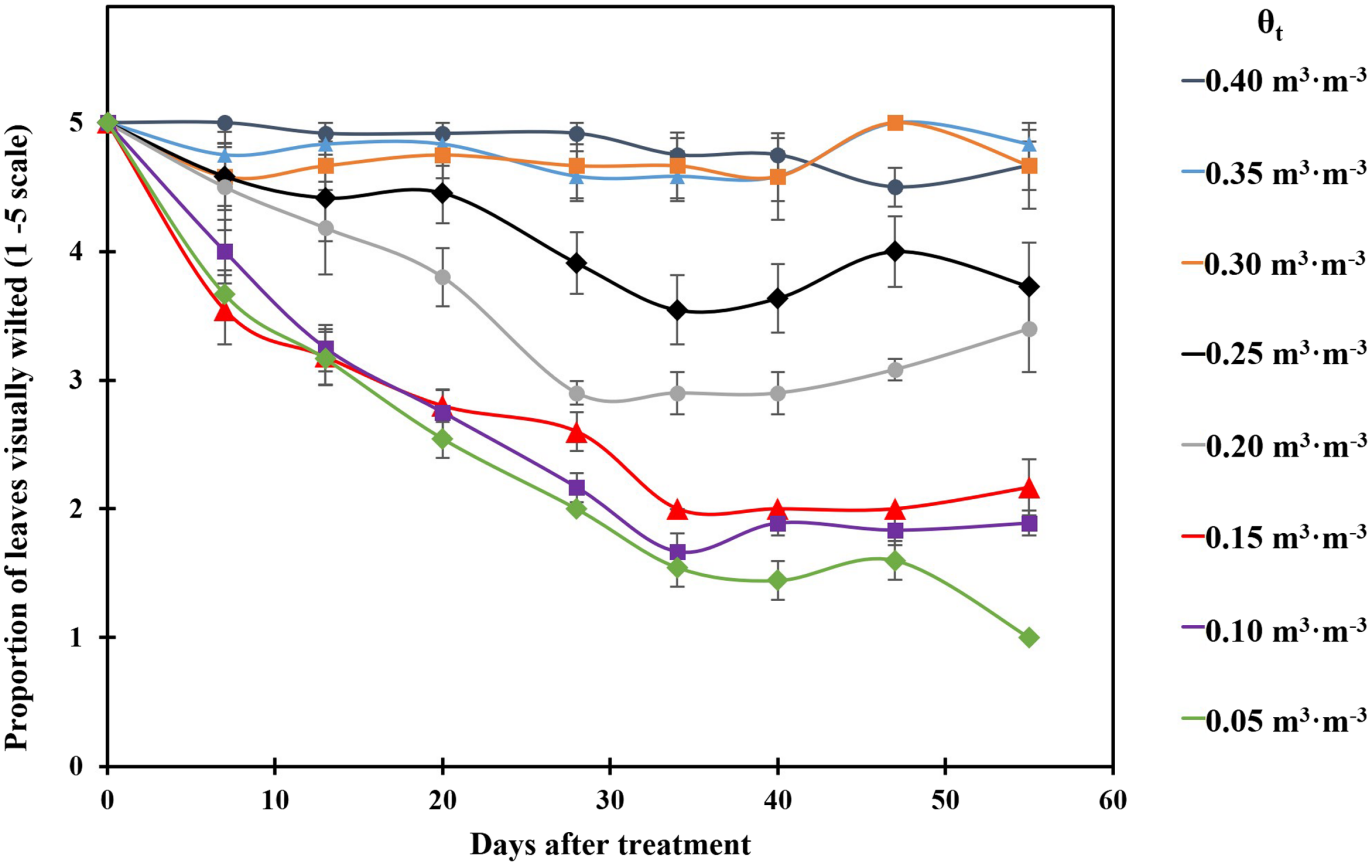
Proportion of visibly wilted leaves of the plants grown at eight substrate volumetric water content treatments (θ_t_) during the experiment. Plants were rated on a scale of 1–5, where 1 = over 65% of the leaves wilted; 2 = 35–65% of the leaves wilted; 3 = up to 35% of the leaves wilted; 4 = less than 10% of the leaves wilted; 5 = plant was fully turgid (Zollinger et al., 2006). Error bars represent SEs of 12 plants.

**Figure 3:**
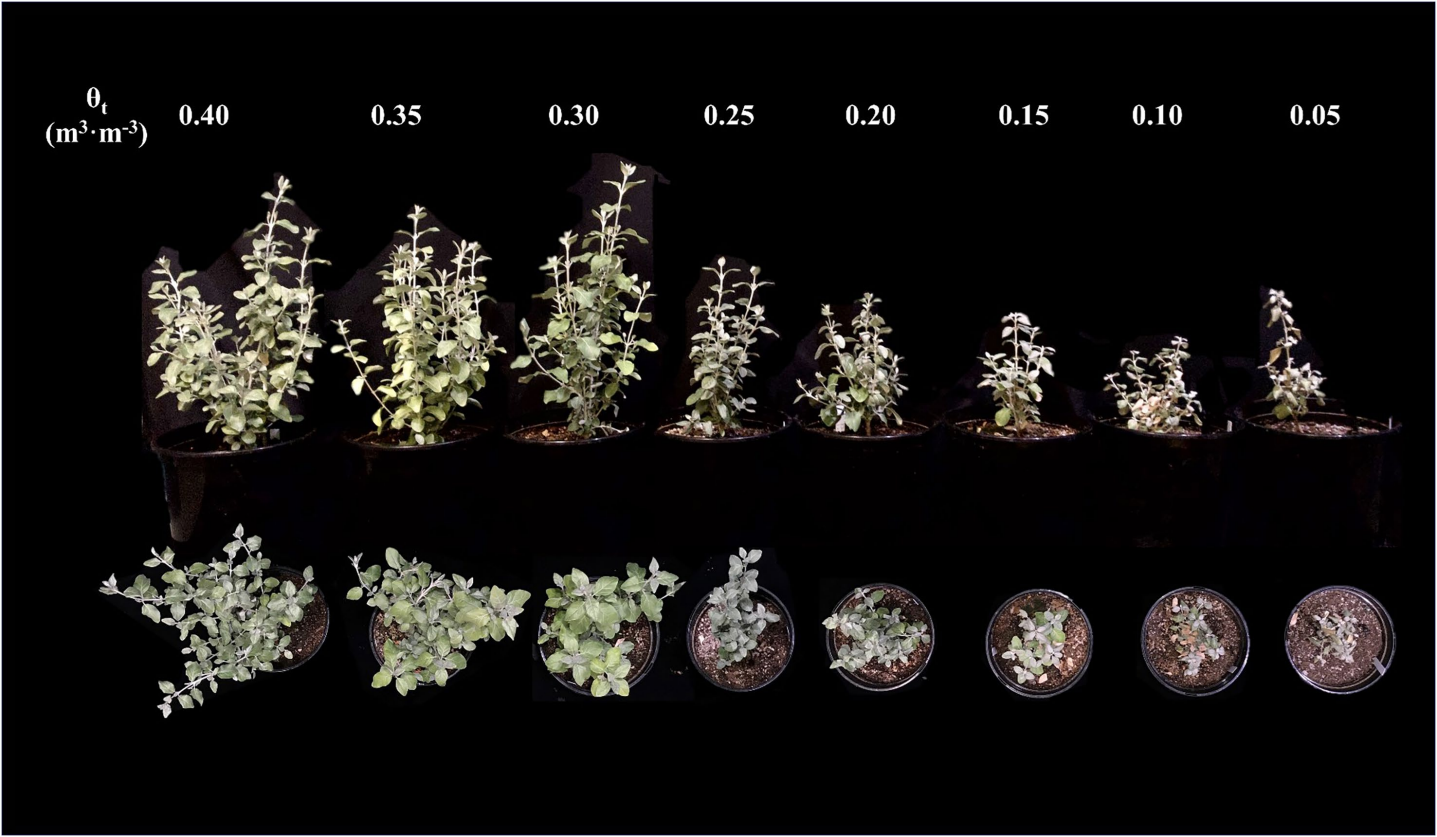
*Shepherdia ×utahensis* plants at eight substrate volumetric water content treatments (θ_t_) at the end of the experiment (photo taken on 10 January 2021).

The proportion of visibly wilted leaves increased at the substrate volumetric water content of 0.25 m^3^·m^−3^ or lower during the experiment (Figure 2). At the termination of the experiment, over 35% of leaves wilted on the plants grown at the substrate volumetric water contents of 0.15, 0.10, and 0.05 m^3^·m^−3^, and the proportion of visibly wilted leaves increased as substrate volumetric water content decreased (Table 2; Figure 2). Plants grown at the substrate volumetric water content of 0.20 m^3^·m^−3^ or higher had acceptable visual quality as their ratings were greater than 3 (Table 2; Figure 3). Plant mortality decreased from 58 to 8% when the substrate volumetric water content increased from 0.05 m^3^·m^−3^ to 0.25 m^3^·m^−3^, and no plants died when substrate volumetric water content was higher than 0.25 m^3^·m^−3^ (Supplementary Figure S1). Greater plant growth indices were observed in *S. ×utahensis* plants at higher substrate volumetric water content (Table 2; Figure 3). Substrate volumetric water content also had effects on relative chlorophyll content, as SPAD values increased with increasing substrate volumetric water content (Table 2). The leaf and stem water contents increased when the substrate volumetric water content increased from 0.05 to 0.40 m^3^·m^−3^. Stem water potential reduced from −0.82 to −1.97 MPa when substrate volumetric water content declined from 0.40 m^3^·m^−3^ to 0.10 m^3^·m^−3^. However, leaf curling indices decreased along with increasing substrate volumetric water content. Cubic curvilinear relationships were found between substrate volumetric water content and proportion of visibly wilted leaves, plant growth indices, SPAD, leaf and stem water contents, stem water potential, or leaf curling indices (Table 2).

**Table 2:** Degree of leaves visibly wilted, plant growth index (PGI), relative chlorophyll content [Soil Plant Analysis Development (SPAD) value], water content of leaves and stems, stem water potential (ψ_stem_), and leaf curling index of *Shepherdia ×utahensis* at eight substrate volumetric water content treatments (θ_t_).

				Water content^y^			
θ_t_ (m^3^·m^−3^)	Leaves wilted (1–5 scale)^z^	PGI^y^	SPAD	Leaf (%)	Stem (%)	ψ_stem_ (MPa)	Leaf curling index^x^
0.40	4.7 ab^v^	33.4 a	58.9 a	59.2 a	62.7 a	−0.82 ab	0.04 bc
0.35	4.8 a	33.2 a	58.2 a	62.6 a	63.6 a	−0.65 a	0.05 bc
0.30	4.7 ab	36.7 a	58.3 a	59.7 a	60.4 a	−0.90 ab	0.02 c
0.25	3.7 bc	23.1 b	56.1 ab	58.1 a	58.5 ab	−1.95 bc	0.06 bc
0.20	3.4 cd	22.6 b	52.1 ab	57.1 a	58.7 ab	−1.45 b	0.03 bc
0.15	2.2 de	17.2 b	49.0 abc	51.1 a	49.9 b	−1.98 c	0.10 abc
0.10	1.9 e	18.3 b	43.4 bc	51.2 a	52.4 b	−1.97 bc	0.11 ab
0.05	1.0 e	17.6 b	34.9 c	23.3 b	36.8 c	−5.76^w^	0.17 a
Linear	**^u^	NS	NS	NS	NS	NS	NS
Quadratic	NS	NS	NS	***	***	NS	NS
Cubic	****	****	****	****	****	*	****

z 1 = over 65% of the leaves wilted; 2 = 35–65% of the leaves wilted; 3 = up to 35% of the leaves wilted; 4 = less than 10% of the leaves wilted; and 5 = plant was fully turgid (Zollinger et al., 2006).

y Plant growth index = [(height + length + width)/3], while water content of leaves and stems was calculated using the equation: [fresh weight (FW) − dry weight (DW)/FW x 100% (Zhou et al., 2021).

x Leaf curling index was determined using the equation: [distance between the margins of flattened leaf (D_max_)-distance between the margins of curling leaf (D_i_)]/D_max_ (Nilsen, 1987).

W Only two data were recorded due to high plant mortality.

v Means with same lowercase letters within a column are not significantly different among treatments by Tukey–Kramer method with a significance level specified at 0.05.

u NS, *, **, ***, **** Nonsignificant, significant at *p* ≤ 0.05, 0.01, 0.001, or 0.0001, respectively.

*Shepherdia ×utahensis* grown at lower substrate volumetric water contents had fewer leaves and shoots (Table 3). Plants also had less total leaf area and dry weight when substrate volumetric water contents decreased from 0.40 to 0.05 m^3^·m^−3^. However, decreasing substrate volumetric water content increased the root-to-shoot ratios. As substrate dried down from 0.40 to 0.05 m^3^·m^−3^, SLA and leaf size declined from 72.1 to 59.7 cm^2^·g^−1^ and 1.97 to 0.97 cm^2^·g^−1^, respectively. In addition, nodule number decreased with decreasing substrate volumetric water content, and nodules were not found on the plants at the substrate volumetric water content of 0.05 m^3^·m^−3^. Cubic curvilinear relationships were observed between substrate volumetric water content and the number of leaves and shoots, leaf area, total DW, root-to-shoot ratio, specific leaf area, leaf size, or the number of nodules (Table 3). In addition, a positive or negative correlation was found between stem water potential, leaf size, and leaf curling index (all *r*^2^ ≥ 0.17, all *p* ≤ 0.02; Supplementary Figure S2).

**Table 3:** Number (no.) of leaves and shoots, total leaf area and dry weight (DW), root to shoot ratio (R/S), specific leaf area (SLA), leaf size, and nodule no. of *Shepherdia ×utahensis* at eight substrate volumetric water content treatments (θ_t_) at the termination of the experiment.

θ_t_ (m^3^·m^−3^)	Leaf no.	Shoot no.	Leaf area (cm^2^)	DW (g)^z^	R/S (g·g^−1^)^y^	SLA (cm^2^·g^−1^)^x^	Leaf size (cm^2^)^w^	Nodule no.
0.40	390.5 ab^v^	13.1 abc	776.1 ab	25.8 ab	0.48 c	72.1 a	1.97 ab	65.6 ab
0.35	438.5 a	15.7 ab	882.5 a	26.4 a	0.47 c	83.6 a	2.06 a	72.4 a
0.30	434.4 a	20.7 a	987.8 a	30.1 a	0.44 c	76.8 a	2.18 a	35.3 bc
0.25	259.2 bc	9.8 bc	414.3 c	17.8 bc	0.90 ab	68.2 a	1.54 abc	37.0 abc
0.20	274.6 bc	9.1 bc	477.7 bc	17.8 bc	0.74 bc	72.2 a	1.71 abc	18.5 c
0.15	126.6 d	6.0 bc	205.8 c	10.2 c	1.04 ab	66.8 a	1.47 bc	1.4 c
0.10	156.1 cd	7.6 bc	208.3 c	12.0 c	1.15 a	67.7 a	1.37 bc	6.3 c
0.05	115.3 d	5.4 c	109.3 c	8.0 c	1.15 ab	59.7 b	0.97 c	0.0 c
Linear	NS^u^	NS	NS	NS	NS	NS	NS	***
Quadratic	NS	NS	NS	NS	NS	NS	NS	NS
Cubic	****	****	****	****	****	**	****	****

z Total DW was the sum of the DW of stems, leaves, and roots.

y Root-to-shoot ratio was calculated using the DW of roots and shoots (leaves and stems).

x SLA was calculated as the ratio of leaf area to leaf DW.

w Leaf size of each plant was calculated as the ratio of total leaf area to the leaf no.

v Means with same lowercase letters within a column are not significantly different among treatments by Tukey–Kramer method with a significance level specified at 0.05.

NS, **, ***, **** Nonsignificant, significant at *p* ≤ 0.01, 0.001 or 0.0001, respectively.

### Gas Exchange

Decreased substrate volumetric water contents resulted in an increase in leaf-to-air VPD (Table 4), which was 2.12 kPa at 0.40 m^3^·m^−3^ but became 3.16 kPa at 0.05 m^3^·m^−3^. When substrate volumetric water contents increased from 0.05 to 0.40 m^3^·m^−3^, stomatal conductance increased from 0.03 to 0.66 mol·m^−2^·s^−1^. Similarly, transpiration rate increased from 0.9 to 9.4 mmol·m^−2^·s^−1^ when substrate volumetric water contents increased from 0.05 to 0.40 m^3^·m^−3^. The net assimilation rate of *S. ×utahensis* ranged from 0.1 to 11.7 μmol·m^−2^·s^−1^ as the substrate volumetric water content increased from 0.05 to 0.40 m^3^·m^−3^. Cubic curvilinear relationships were observed between substrate volumetric water content and VPD, stomatal conductance, transpiration rate, or net assimilation rate (Table 4).

**Table 4:** Leaf-to-air vapor pressure deficit (VPD), stomatal conductance (*g_s_*), transpiration rate (*E*), and net assimilation rate (P_n_) of *Shepherdia ×utahensis* at eight substrate volumetric water content treatments (θ_t_).

θ_t_ (m^3^·m^−3^)	VPD (kPa)	*g_s_* (mol H_2_O·m^−2^·s^−1^)	*E* (mmol H_2_O·m^−2^·s^−1^)	P_n_ (μmol CO_2_·m^−2^·s^−1^)
0.40	2.12 b^z^	0.66 ab	9.4 ab	11.7 ab
0.35	1.87 b	0.80 a	11.0 a	13.4 a
0.30	1.79 b	0.83 a	10.8 a	14.6 a
0.25	2.39 ab	0.38 bc	7.1 bc	8.9 bc
0.20	2.05 b	0.61 ab	8.9 ab	11.6 ab
0.15	2.96 a	0.17 c	4.3 cd	5.2 bcd
0.10	2.93 a	0.10 c	3.0 d	4.0 cd
0.05	3.16 a	0.03 c	0.9 d	0.1 d
Linear	NS^y^	NS	NS	NS
Quadratic	NS	NS	NS	NS
Cubic	****	****	****	****

z Means with same lowercase letters within a column are not significantly different among treatments by Tukey–Kramer method with a significance level specified at 0.05.

NS, **** Nonsignificant, significant at *p* ≤ 0.0001, respectively.

### Leaf Trichomes and Fine-Scale Morphology

**Figure 4:**
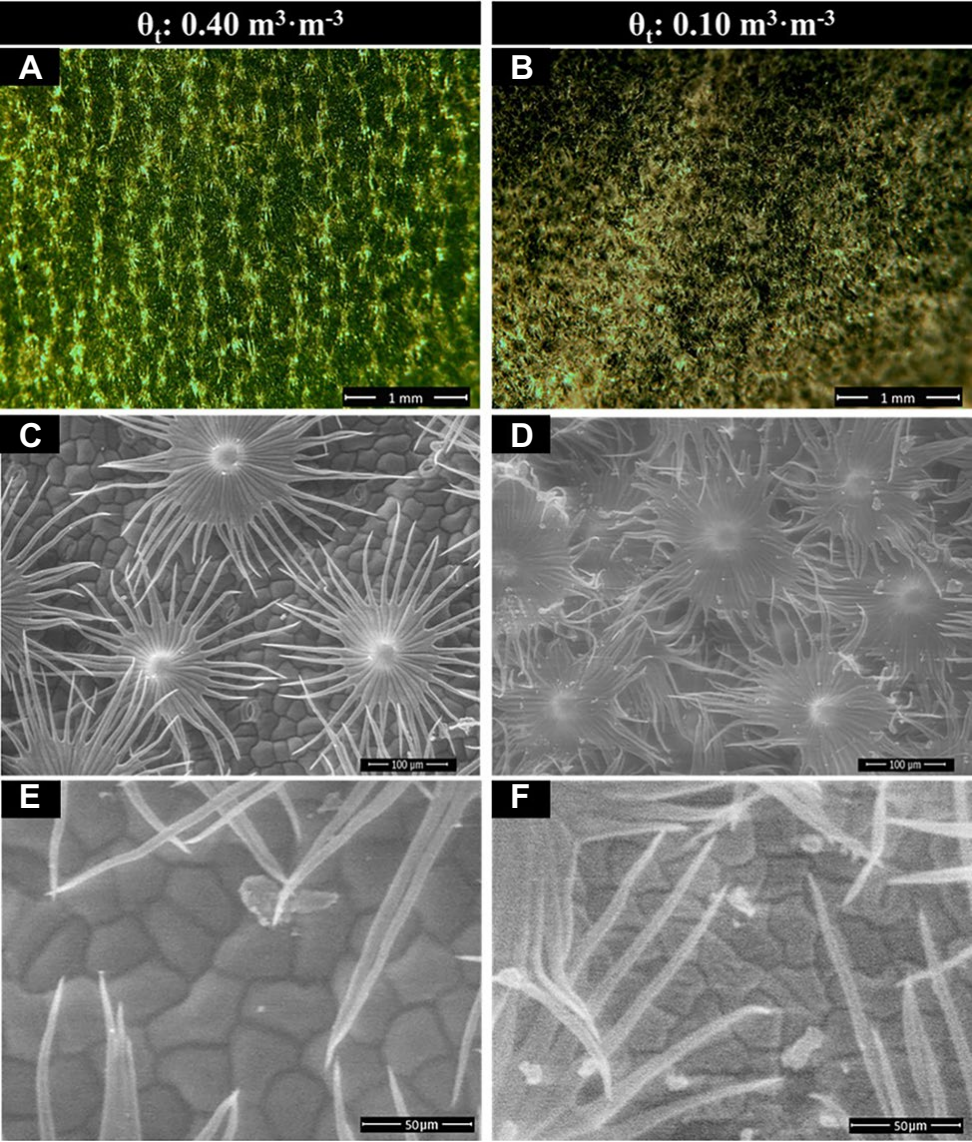
Dissected compound microscopy image of the leaf upper (adaxial) surface **(A,B)** and scanning electron microscopy (SEM) image of leaf trichomes **(C,D)** and epidermal cells **(E,F)** of *Shepherdia ×utahensis* plants at the substrate volumetric water content treatments (θ_t_) of 0.40 and 0.10 m^3^·m^−3^.

**Figure 5:**
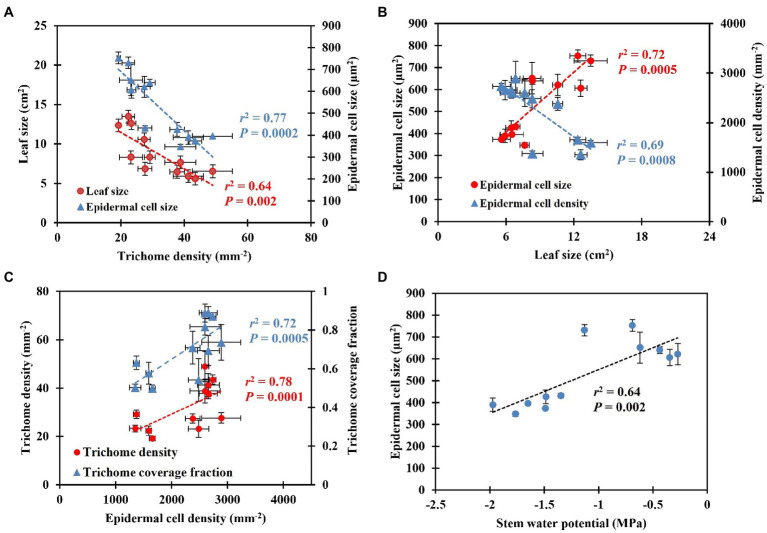
Correlation between leaf size, epidermal cell size, and trichome density **(A)**, epidermal cell size and density and leaf size **(B)**, trichome density, trichome coverage fraction, and epidermal cell density **(C)**, epidermal cell size and stem water potential **(D)**. The error bars represent the SEs of three leaves sampled from each plant.

*Shepherdia ×utahensis* became silvery when grown in drier substrates toward the end of the experiment (Figures 4A,B). Higher trichome density and smaller epidermal cells were observed on the leaves of plants grown at the substrate volumetric water content of 0.10 m^3^·m^−3^ than those at 0.40 m^3^·m^−3^ (Figures 4C–F). Decreasing water content in substrate linearly increased leaf trichome density, trichome coverage fraction, and epidermal cell density (Table 5). Nonetheless, uncovered stomata, trichome radius, and epidermal cell size decreased linearly as substrate volumetric water contents decreased. Trichome density was negatively influenced by epidermal cell size and leaf size (Figure 5A), and the total number of trichomes per leaf was similar among plants at different substrate volumetric water contents (*p* = 0.97; Supplementary Figure S3). Leaf size also increased with increasing epidermal cell size or decreasing epidermal cell density (Figure 5B) but was not affected by the total number of epidermal cells (*p* = 0.19; Supplementary Figure S3). Positive correlation showed between the density of trichomes and epidermal cells and trichome coverage fraction (Figure 5C), and plants had a similar trichome-to-epidermal cell ratio at various substrate volumetric water contents (*p* = 0.34; Supplementary Figure S3). Epidermal cell size was positively correlated with stem water potential (Figure 5D). In addition, a negative correlation was observed between trichome density and trichome radius (*r*^2^ = 0.79, *p* < 0.0001; Supplementary Figure S3).

**Table 5:** Fine-scale morphology and leaf reflectance at the wavelengths of photosynthetically active radiation (PAR), blue, green, and red light of *Shepherdia ×utahensis* at the substrate volumetric water content treatments (θ_t_) of 0.40, 0.30, 0.20, and 0.10 m^3^·m^−3^.

		Fine-scale morphology					Leaf reflectance^x^			
θ_t_ (m^3^·m^−3^)	Trichome density (mm^−2^)	Trichome coverage fraction^z^	Uncovered stomata (mm^−2^)	Trichome radius (μm)	Epidermal cell size^y^ (μm^2^)	Epidermal cell density (mm^−2^)	PAR	Blue	Green	Red
							%			
0.40	23.9 b^w^	0.54 b	29.1 ab	195 a	666 a	1544 b	12.9 b	11.6 b	17.5 b	10.9 b
0.30	24.3 b	0.61 ab	34.0 a	193 a	668 a	1539 b	14.0 ab	11.7 ab	18.8 ab	11.9 ab
0.20	34.7 ab	0.81 a	12.9 bc	164 b	402 b	2583 a	16.1 ab	15.0 a	19.3 ab	14.6 ab
0.10	44.6 a	0.82 a	10.0 c	137 b	386 b	2662 a	18.8 a	17.5 a	22.5 a	17.4 a
Linear	***^v^	**	**	****	****	****	**	**	*	**
Quadratic	NS	NS	NS	NS	NS	NS	NS	NS	NS	NS
Cubic	NS	NS	*	NS	**	**	NS	NS	NS	NS

z Trichome coverage fraction = (area covered by trichomes)/(total image area).

y Epidermal cell size was estimated by measuring the area of eight randomly selected epidermal cells in each image.

x Reflectance of PAR was determined using the wavelengths from 400 to 700 nm, while the reflectance of blue, green, and red light was recorded at the wavelengths of 450, 530, and 660 nm, respectively (Kusuma et al., 2020).

w Means with same lowercase letters within a column are not significantly different among treatments by Tukey–Kramer method with a significance level specified at 0.05.

v NS, *, **, ***, **** Nonsignificant, significant at *p* ≤ 0.05, 0.01, 0.001, or 0.0001, respectively.

**Figure 6:**
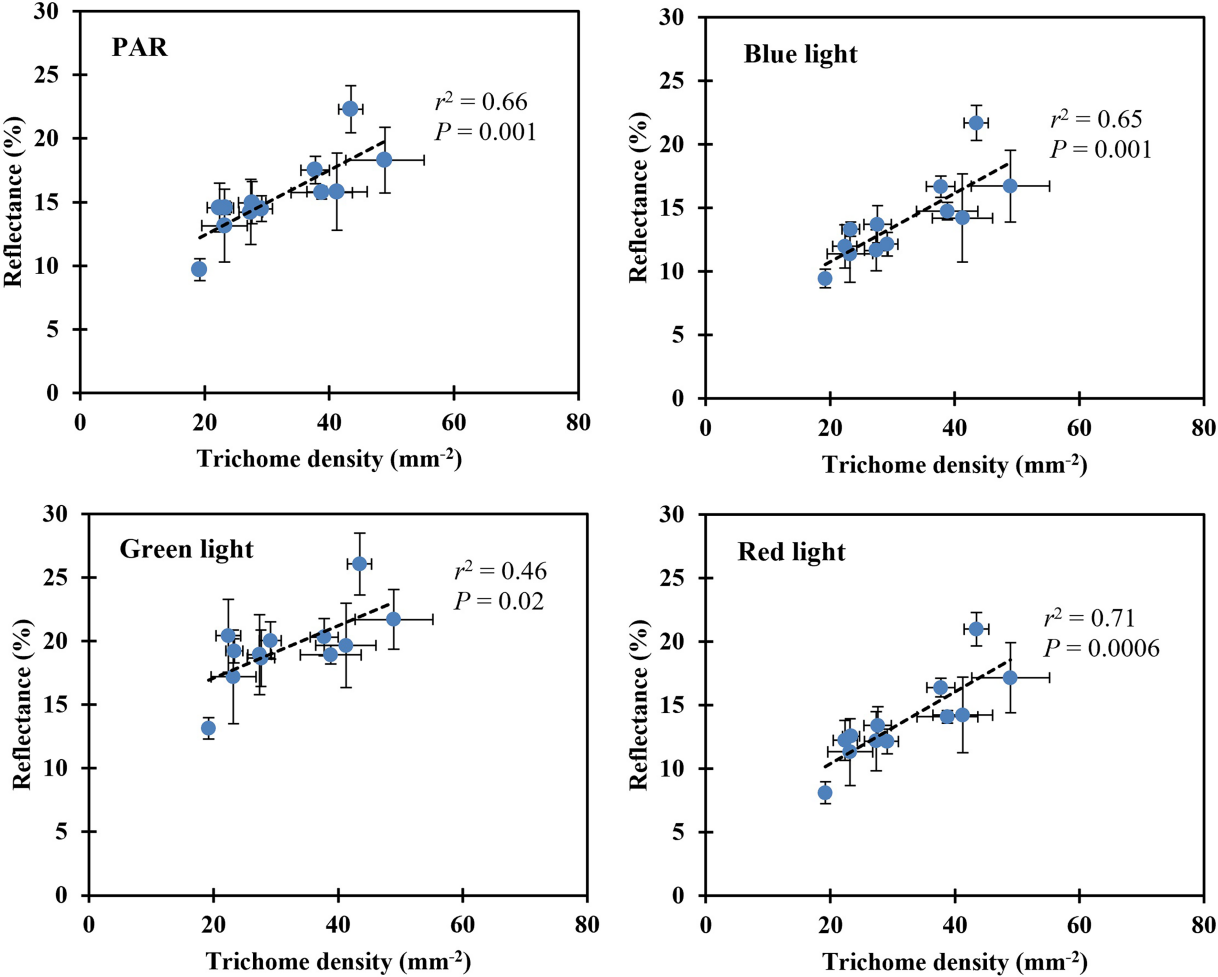
Correlation between the leaf upper (adaxial) surface trichome density and reflectance of PAR, blue, green, and red light. The reflectance of PAR was determined using the mean reflectance between the wavelengths (λ) ranging from 400 to 700 nm. The reflectance of blue, green, and red light was determined using the λ at 450, 530, and 660 nm, respectively (Kusuma et al., 2020). The error bars represent the SEs of three leaves sampled from each plant.

Leaf reflectance of PAR, blue, green, and red light increased linearly with decreasing substrate volumetric water content (Table 5). Leaves reflected 46% more PAR when plants were grown at the substrate volumetric water content of 0.10 m^3^·m^−3^ than those at 0.40 m^3^·m^−3^. In addition, blue-, green-, and red-light reflectance increased by 51, 29, and 60%, respectively, when plants were grown at the substrate volumetric water content of 0.10 m^3^·m^−3^ than those at 0.40 m^3^·m^−3^. The reflectance of PAR, blue-, green-, and red-light correlated positively with trichome density (all *r*^2^ ≥ 0.46, all *p* ≤ 0.02; Figure 6).

## Discussion

Plant morphology and physiology in this study changed along with decreasing substrate matric potential that resulted from reduced substrate volumetric water contents (Table 1). As substrate volumetric water content decreased, *S. ×utahensis* leaves and stems dehydrated, and the proportion of visibly wilted leaves increased (Table 2). In addition, plant growth indices, relative chlorophyll content (SPAD reading), numbers of shoots and leaves, total leaf area and dry weight, and photosynthesis were impaired (Tables 2–4). These results are in line with previous studies that reported negative effects of water stress on aesthetic appearance, plant growth, and net assimilation rate of ornamental plants (Niu et al., 2006; Cai et al., 2012; Zhou et al., 2021). In this case, decreased stem water potential is best interpreted as a passive response resulting from the effects of decreased soil water potential and higher leaf evaporative demand (Table 2). Similarly, *Rosa ×hybrid* (rose) and *Nerium oleander* (oleander) decreased stem water potential in response to low substrate or soil water potential under drought conditions (Molz, 1981; Niu et al., 2006; Cai et al., 2012). Decreased substrate volumetric water contents also inhibited nodule formation in *S. ×utahensis* (Table 3), which suggested that infection of symbiotic actinobacteria was affected by water availability. Actinobacteria move with water in the soil, and the process of reaching and infecting the roots of host plants slows down when soil water content decreases (Huss-Danell, 1997).

Plant morphological and physiological acclimations were observed in this study. In response to drought, *S. ×utahensis* reduced midday stomatal conductance to a value close to 0 when substrate volumetric water content decreased (Table 4). Midday stomatal conductance is positively correlated to stomatal opening and plant water status (Zhang et al., 2013). When *S. ×utahensis* plants dehydrated as a result of decreasing substrate volumetric water contents, plants closed their stomata to reduce transpiration and stomatal conductance as a drought acclimation to maintain plant water status and prevent water losses and further dehydration (Martinez-Vilalta and Garcia-Forner, 2017). Although CO_2_ uptake is limited when stomata are closed and stomatal conductance reduced (Aroca, 2012), plants had a lower proportion of visibly wilted leaves in this study or better aesthetic quality under drought conditions in other reports. These plants were considered drought tolerant in ornamental plant evaluations in semiarid regions in Australia and the United States (Zollinger et al., 2006; Kjelgren et al., 2009; Reid et al., 2017). *Shepherdia ×utahensis* reduced its midday stomatal conductance at lower water availability and can be considered as a low water-use landscape plant. Plants have the capacity of regulating stomatal conductance that is related to their habitat aridity. Kjelgren et al. (2009) reported that plants native to arid regions, such as *Dianella revoluta* ‘Breeze’ (‘Breeze’ blueberry lily) and *Ptilotus nobilis* (yellow tails), showed greater reduction of stomatal conductance compared with those from humid areas.

Because of restricted transpiration, plants with acclimation capability may reduce leaf size to enhance convective heat loss to mitigate heat stress that causes high leaf-to-air VPD and leaf wilting (Tables 3, 4; Nobel, 2009; Devi et al., 2015). The fact that leaf-to-air VPD increased when substrate volumetric water content decreased (Table 4) is likely a direct consequence of increased leaf temperature because leaf vapor pressure is estimated by leaf temperature. To avoid heat stress, leaf energy is balanced primarily using sensible heat loss under drought (Bowen, 1926). The efficacy of sensible heat loss relates to boundary layer resistance, which is positively correlated to leaf width (Nobel, 2009). Under drought conditions, cell division and leaf expansion are limited (Dale, 1988), and smaller leaves are beneficial for dissipating heat through convection and conduction to maintain leaf temperature close to air temperature (Nobel, 2009). In this study, *S. ×utahensis* produced smaller leaves under water stress (Table 3) and leaf size of plants grown at the substrate volumetric water content of 0.05 m^3^·m^−3^ was 51% smaller than those at 0.40 m^3^·m^−3^. This result is in line with previous studies that consistently reported reductions in leaf size under water stress for drought-tolerant ornamental plants (Mee et al., 2003; Zollinger et al., 2006). For instance, Zollinger et al. (2006) suggested that small leaves allow *Lavandula angustifolia* (English lavender) and *Penstemon ×mexicali* ‘Red Rocks’ (‘Red Rocks’ penstemon) to reduce water loss when irrigation intervals were increased from 1 week to 4 weeks. Toscano et al. (2014) also found that leaf size of *Viburnum tinus* ‘Lucidum’ (‘Lucidum’ viburnum) decreased by 19% to acclimate to drought stress.

*Shepherdia ×utahensis* decreased total leaf area under water stress as a result of reductions in leaf number and size (Table 3). However, plants with decreased total leaf area have fewer stomata and less light interception, which controls transpiration and leaf temperature, respectively (Zollinger et al., 2006; Toscano et al., 2014). Reduced total leaf area has been reported as a means of avoiding drought stress in ornamental plants such as *Lavandula angustifolia*, *Pittosporum tobira* (pittosporum), and *Viburnum tinus* ‘Lucidum’ (Zollinger et al., 2006; Toscano et al., 2014). The root growth of *S. ×utahensis* was enhanced at low substrate volumetric water content, while shoot growth was inhibited, resulting in a higher root-to-shoot ratio (Table 3), which helps plants to obtain water more efficiently. *Rosa hybrida* ‘Ferdy’ (‘Ferdy’ rose) and *Populus cathayana* (poplar) have been observed to increase root growth to maintain water status under water stress (Henderson et al., 1991; Yin et al., 2004). Drought-tolerant plants native to the western United States also produce small leaves and deep roots to reduce water demand and loss and increase water uptake (Mee et al., 2003).

In this study, as substrate volumetric water content decreased, leaves of *S. ×utahensis* curled as stem water potential became more negative. At the substrate volumetric water content of 0.05 m^3^·m^−3^, the leaf curling index was 0.17, suggesting that the light interception area was 83% that of flattened leaves. Similarly, *Dianella revoluta* ‘Breeze’ and *Ctenanthe setosa* (prayer plant) have been shown to minimize sunlight exposure through leaf curling under water deficit (Kjelgren et al., 2009; Nar et al., 2009). Although light-harvesting efficiency is reduced, leaf curling limits water loss from transpiration and protects plants from overheating to sustain photosystem functions and other biochemical/physiological processes (Hook and Hanna, 1994; Fitter and Hay, 2002). In addition, as the rooting substrate became drier in this study, specific leaf area decreased, indicating that leaves became thicker (Table 3), which prevented leaves from overheating. Plants may decrease specific leaf area to acclimate to water stress as reported in *Ptilotus nobilis* (Kjelgren et al., 2009).

The trichome density of *S. ×utahensis* in this study was affected by substrate water availability and plant water status (Table 5). Water-stressed *S. ×utahensis* produced densely packed trichomes, resulting in a silvery appearance, while well-watered plants had fewer trichomes to cover epidermal cells and exhibited a greener color (Table 5; Figure 4). Trichomes promote leaf reflectance (Figure 6), which helps balance energy and reduce heat stress (Ehleringer et al., 1976). Positive effects of trichomes on leaf reflectance of visible light have been reported on *Verbascum thapsus* (common mullein) and *Salix commutata* (undergreen willow; Wuenscher, 1970; Mershon et al., 2015). However, because trichomes are broad-spectrum reflectors (Bickford, 2016), the reflectance of PAR, blue, green, and red light is proportional to the trichome density (Figure 6). When substrate volumetric water content decreased, the reflectance of green light (530 nm) did not increase as much as blue light (450 nm) and red light (660 nm) due to the chlorophyll in the epidermal cells (Table 5). Increased leaf reflectance has been shown to sacrifice the efficacy of light-harvesting pigments (Bickford, 2016) and reduce the net assimilation rate when plants are grown in drier conditions. Previous research also suggested that trichomes improved the reflectance of near-infrared light (Ehleringer, 1988). However, in this study, denser trichomes produced in drier substrate did not affect near-infrared light reflectance of *S. ×utahensis* (Supplementary Figure S4). Slaton et al. (2001) reported similar results that near-infrared light reflectance was not affected by increased trichome density in 48 species. More studies are needed to evaluate the effects of trichomes on near-infrared reflectance.

Increased trichome density has smaller effects on decreasing gas exchange coed with the effects on leaf reflectance (Jordan et al., 2005; Moles et al., 2020). However, densely packed trichomes covering the stomata of *S. ×utahensis* may increase resistance to transpiration and reduce water loss (Table 5; Figure 4D). Leaf trichomes also increase leaf roughness and increase the laminar boundary layer to restrict air movement across leaf surfaces to reduce transpiration (Grammatikopoulos and Manetas, 1994). *Eriogonum corymbosum* (crisp-leaf buckwheat) and *S. rotundifolia* produce leaf trichomes for better protection from wind and to maintain water status (Miller, 2011). Densely packed trichomes add an atmospheric boundary layer that imposes additional resistance to water vapor diffusion (Nobel, 2009). However, CO_2_ influx is also limited by the boundary layer resistance, decreasing the net assimilation rate (Ehleringer et al., 1976). Although trichome-induced boundary layer resistance has a smaller effect on transpiration than stomatal conductance (Parkhurst, 1976), it still provides an advantage for desert plants to survive in dry and hot conditions.

Trichome density changes genetically (adaptation) and environmentally (acclimation). The genetic regulation of trichome density of *Caragana korshinskii* (Korshinsk pea shrub) has been reported by Ning et al. (2016). However, it is unclear how xeric plants change their trichome density to acclimate to drought conditions. A negative correlation between leaf trichome density and leaf size or epidermal cell size occurred in this study (Figure 5A), which suggests that cell expansion may control trichome density. Low trichome coverage fraction, which was related to greater space between trichomes, showed when epidermal cell density decreased, indicating cell expansion may coordinate trichome density. Ascensão and Pais (1987) reported the number of trichomes is determined during leaf lifespan, and leaf cell differentiation does not affect trichome number. Similar results showed in our research that plants had similar total numbers of trichomes per leaf at different substrate volumetric water contents. This may indicate that *S. ×utahensis* develops trichomes independent of leaf development. In fact, trichomes develop at the early stage of leaf development and often earlier than stomatal development (Werker, 2000). For instance, trichomes of *Inula viscosa* (false yellowhead) are fully developed and reach mature size when leaves are 2 mm long; however, a mature leaf is 6–8 cm long (Werker and Fahn, 1981). *Ocimum basilicum* (basil) forms trichomes at an early stage of leaf development and trichomes then grow independently (Werker et al., 1993). In the same study, trichomes covered young leaves but became more widely spaced when leaf cells started to expand (Werker et al., 1993). In our study, the total number of epidermal cells per leaf was similar on plants at different substrate volumetric water contents, which indicates cell differentiation might have minor effects on regulating trichome density. In contrast, cell expansion might be the main factor for regulating trichome density because leaf size, epidermal cell size, and the space among trichomes changed along with substrate volumetric water contents and correlated significantly with trichome density of *S. ×utahensis* (Figures 5A,B). Ehleringer (1982) found a negative correlation between leaf size and trichome density of *Encelia farinosa*, but cell size was not determined. Cell enlargement at high soil moisture levels amplified leaf size and the space among trichomes, reducing the trichome density on the *S. ×utahensis* leaves in this study. The relationships between trichome density, epidermal cell size and density, and leaf reflectance might indicate changes in cell size predominantly controls trichome density to modify leaf reflectance.

Modifying leaf reflectance via the change in cell size helps rapidly acclimate to environmental change without compromising whole leaf function (Brodribb et al., 2013). Cell-expansion-driven leaf anatomic change has been widely reported on adjusting stomatal density (Xu and Zhou, 2008; Murphy et al., 2016). For instance, Murphy et al. (2016) observed that cell expansion was the predominant factor for coordinating vein and stomata density of eight angiosperm species under sun and shade. Stomatal density decreases and the size of guard cells increases when leaf water potential increases (Xu and Zhou, 2008), suggesting cell expansion not only enlarges the distance between epidermal appendages but also increases their size.

Environmental factors also promote leaf trichome density. Such factors include increased leaf-to-air VPD (Ehleringer, 1982; Shibuya et al., 2009, 2016) and drought (Quarrie and Jones, 1977; Banon et al., 2004), all of which negatively affect plant water status. For instance, high leaf-to-air VPD may increase water loss via transpiration, leading to plant dehydration. Leaf trichome density of *Cucumis sativus* increased when air humidity decreased from 90 to 20% at 28°C, causing leaf-to-air VPD to increase from 0.4 to 3.0 kPa (Shibuya et al., 2016). Shibuya et al. (2016) did not investigate cell or leaf expansion of *C. sativus*, but increased leaf-to-air VPD may promote trichome density because rising leaf-to-air VPDs reduces cell size (Murphy et al., 2014), making space between trichomes smaller. In this study, higher leaf-to-air VPD and smaller leaves were observed when *S. ×utahensis* plants grew at the lower θ_t_ and the smaller epidermal cell size resulted in greater trichome density. Therefore, because increased leaf-to-air VPD and drought led to a reduction in cell enlargement and denser trichomes in *S. ×utahensis*, leaf trichome density was regulated using turgor-pressure-driven cell expansion to acclimate to drought conditions.

## Conclusion

As the soilless substrate became drier, *S. ×utahensis* exhibited poorer visual quality due to wilted foliage. Water stress also imposed negative effects on plant growth and gas exchange. When substrate water levels decreased, *S. ×utahensis* increased root growth to increase the ability to uptake water. Stressed plants also lowered total leaf area and stomatal conductance to reduce water loss via transpiration. Leaf temperature was regulated through smaller leaves, curled leaves, and densely packed trichomes. Substrate volumetric water content and stem water potential negatively affected trichome density, which helped reflect a broad spectrum of visible light under drought. An increase in cell size and leaf expansion may have regulated the trichome density. Under water stress, dense trichomes resulted from the limited cell expansion and small space between trichomes. In contrast, greater water availability increased cell size which promoted cell/leaf expansion and enlarged trichome size and the space between trichomes, leading to lower trichome density and improve light-harvesting efficiency.

## Data Availability Statement

The original contributions presented in the study are included in the article/Supplementary Material, further inquiries can be directed to the corresponding authors.

## Author Contributions

J-JC and YS conceptualized and designed the study and drafted the manuscript. J-JC performed the experiment and data analysis. KK, LO, SJ, and LH reviewed and edited the manuscript. All authors contributed to the article and approved the submitted version.

## Funding

This research was supported in part by the United States Department of Agriculture (USDA) National Institute of Food and Agriculture (NIFA) Hatch project UTA01381, New Faculty Start-Up Funds from the Office of Research and Graduate Studies, the Center for Water-Efficient Landscaping, and the Utah Agricultural Experiment Station, Utah State University, and approved as journal paper number 9568. In addition, funding for this project was made possible by the USDA’s Agricultural Marketing Service through grant AM190200XXXXG005 [19-1044-110-SF] and grant AM170100XXXXG022 [190905].

## Conflict of Interest

The authors declare that the research was conducted in the absence of any commercial or financial relationships that could be construed as a potential conflict of interest.

## Publisher’s Note

All claims expressed in this article are solely those of the authors and do not necessarily represent those of their affiliated organizations, or those of the publisher, the editors and the reviewers. Any product that may be evaluated in this article, or claim that may be made by its manufacturer, is not guaranteed or endorsed by the publisher.
