# Supplementary material for: Effects of Water Availability on Leaf Trichome Density and Plant Growth and Development of Shepherdia ×utahensis
Source: Front Plant Sci. 2022 May 18;13:855858. doi: 10.3389/fpls.2022.855858 (PMC9158747; doi:10.3389/fpls.2022.855858)
Supplement: Supplementary file 1 [file Data_Sheet_1.docx]

**Supplemental Materials**

**
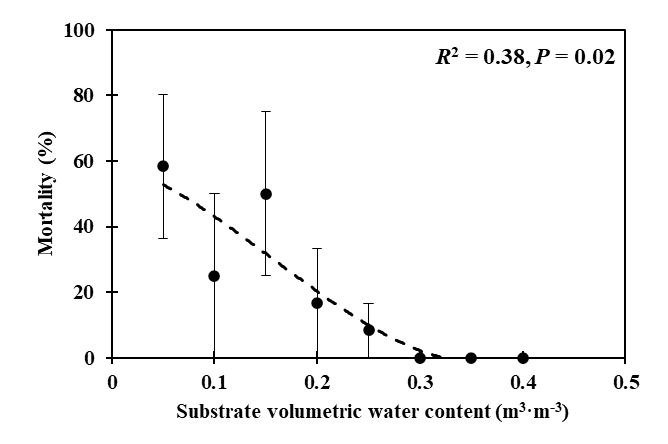
**

Figure S1. Plant mortality at eight substrate volumetric water content treatments (θ_t_) at the termination of the experiment. The error bars represent the standard errors of three blocks (replicates), four plants in each block and treatment.


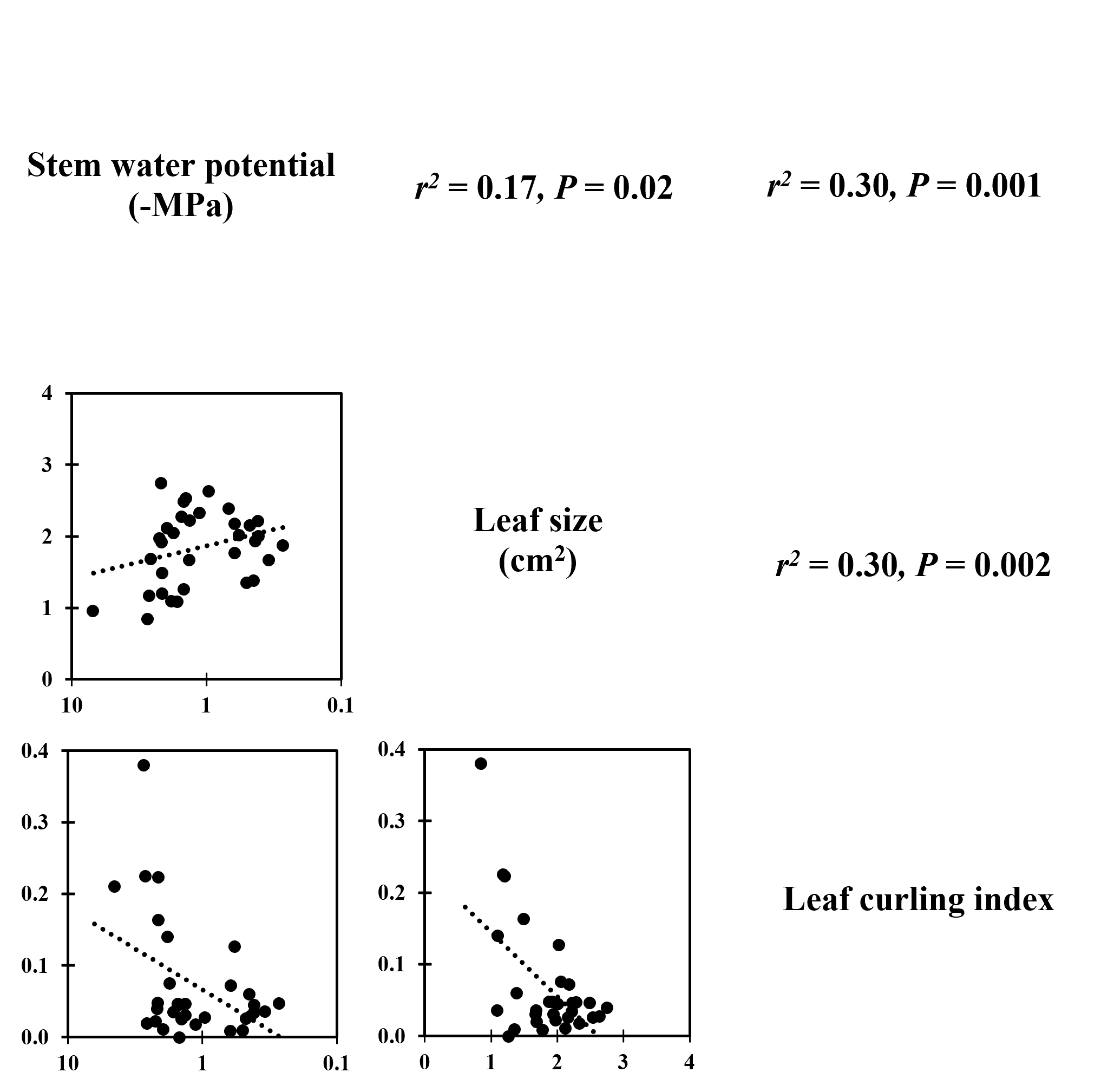


Figure S2. Correlation between stem water potential, leaf size, and leaf curling index at the termination of the experiment. Leaf size of each plant was calculated as the ratio of total leaf area to the number of leaves. Leaf curling index was determined using the equation: [distance between the margins of flattened leaf (Dmax)-distance between the margins of curling leaf (Di)]/Dmax (Nilsen, 1987).


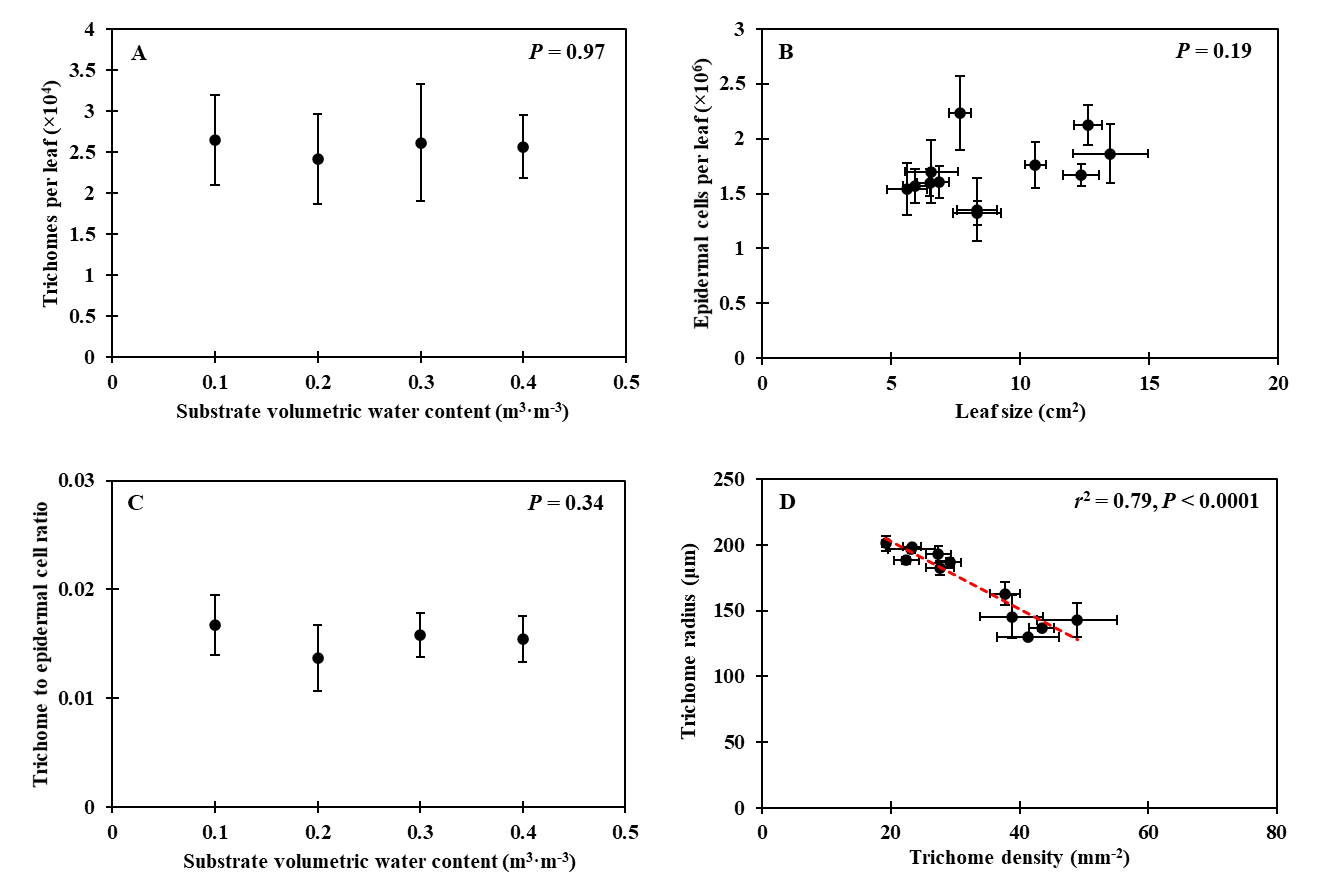


Figure S3. Total number of trichomes per leaf (A) and trichome to epidermal cell ratio (C) at different substrate volumetric water contents, correlation between total number of epidermal cells per leaf and leaf size (B), correlation between trichome density and trichome radius (D). The error bars represent the standard errors of three plants in (A) and (C), while the error bars represent the standard errors of three leaves sampled from each plant in (B) and (D).


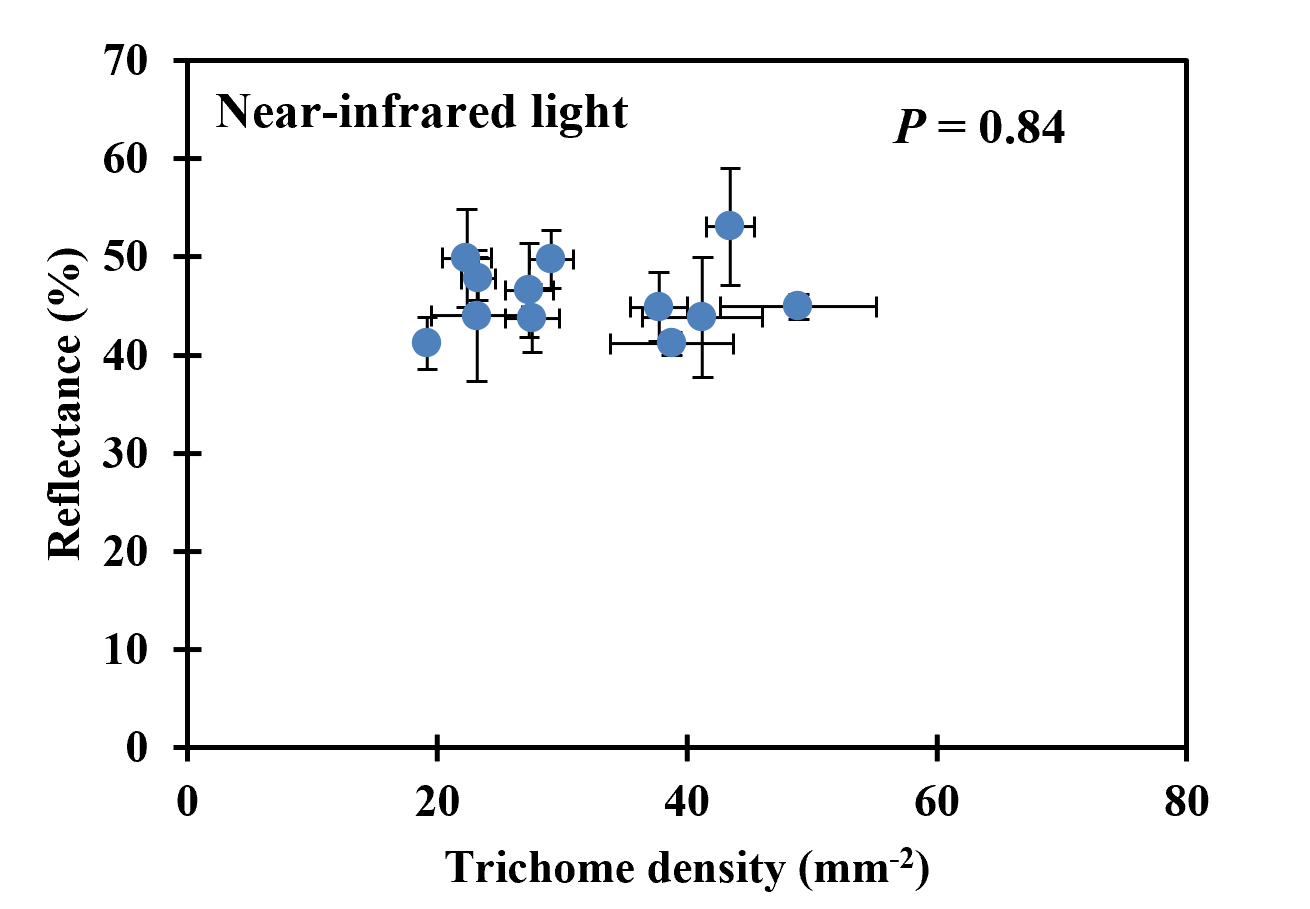


Figure S4. Correlation between near-infrared light reflectance and trichome density on the leaf upper (adaxial) surface. The reflectance of near-infrared light was determined using the wavelength at 730 nm (Kusuma et al., 2020). The error bars represent the standard errors of three leaves sampled from each plant.
